# Pseudohypoaldosteronism type 1 and Liddle’s syndrome mutations that affect the single-channel properties of the epithelial Na^+^ channel

**DOI:** 10.14814/phy2.12600

**Published:** 2015-11-04

**Authors:** Nina Boiko, Volodymyr Kucher, James D Stockand

**Affiliations:** Department of Physiology, University of Texas Health Science CenterSan Antonio, Texas

**Keywords:** Degenerin, distal nephron, hypertension, renal sodium excretion

## Abstract

These studies test whether three disease-causing mutations in genes (*SCNN1A and SCNN1G*) encoding subunits of the epithelial Na^+^ channel, ENaC, affect the biophysical and gating properties of this important renal ion channel. The S562P missense mutation in *α*ENaC and the K106_S108delinsN mutation in *γ*ENaC are associated with pseudohypoaldosteronism type 1 (PHA1). The N530S missense mutation in *γ*ENaC causes Liddle’s syndrome. Incorporation of S562P into *α*ENaC and K106_S108N into *γ*ENaC resulted in significant decreases in macroscopic ENaC currents. Conversely, incorporation of N530S into *γ*ENaC increased macroscopic ENaC current. The S562P substitution resulted in a nonfunctional channel. The K106_S108N mutation produced a functional channel having a normal macroscopic current–voltage relation, there was a slight but significant decrease in unitary conductance and a marked decrease in single-channel open probability. The N530S substitution increased single-channel open probability having no effect on the macroscopic current–voltage relation or unitary conductance of the channel. These findings are consistent with mutation of residues at 562 in *α*ENaC and 530 in *γ*ENaC, and a 3′ splice site in *SCNN1G* (318-1 G→A; K106_108SdelinsN) resulting in aberrant ENaC activity due to changes in the biophysical and gating properties of the channel. Such changes likely contribute to the cellular mechanism underpinning the PHA1 and Liddle’s syndrome caused by these mutations in ENaC subunits.

## Introduction

The epithelial Na^+^ channel, ENaC, is expressed in the kidney in the apical membrane of principal cells. The activity of ENaC in these cells is limiting for transcellular Na^+^ reabsorption across the aldosterone-sensitive distal nephron (ASDN). This ENaC-mediated Na^+^ reabsorption protects vascular volume and thus, blood pressure. Consequently, ENaC is an important end-effector of the renin-angiotensin-aldosterone system (RAAS). The activation of ENaC by aldosterone decreases urinary Na^+^ and volume excretion contributing to feedback regulation of blood pressure by the RAAS.

Gain of function mutations in ENaC cause Liddle’s syndrome: An autosomal dominant hypertension presenting with low plasma renin activity, hypokalemia, and metabolic alkalosis (Hansson et al. 1995; Schild et al. 1995; Snyder et al. 1995). Loss of function mutations in ENaC cause pseudohypoaldosteronism type 1 (PHA1): An autosomal recessive renal salt wasting associated with elevated plasma renin activity, hyperkalemia, and metabolic acidosis (Hummler et al. 1997; Hummler and Horisberger 1999; Bonny and Hummler 2000). Such observations emphasize the important function performed by ENaC in the human kidney.

ENaC is a member of the Degenerin/ENaC ion channel family (Canessa et al. 1993, 1994; Lingueglia et al. 1993). Every multicellular eukaryote species expresses Deg/ENaC channels (Stockand 2015). Dissimilar to many other members of the Deg/ENaC ion channel family, ENaC is primarily expressed in (Na^+^-transporting) epithelial cells rather than neurons (Kellenberger and Schild 2002; Rossier et al. 2002). During evolution, ENaC first appears with the emergence of air breathing animals and terrestrial life (Stockand 2015). In the lungs, ENaC function is necessary for fluid clearance from aveolar spaces. This is a requirement for air breathing. In the kidneys, as mentioned above, ENaC function influences plasma volume by fine-tuning renal Na^+^ excretion through control of Na^+^ reabsorption across the distal nephron.

The structure of Deg/ENaC channels was revealed by the crystallization of chicken acid-sensing ion channel 1 (ASIC1; Baconguis and Gouaux 2012; Baconguis et al. 2013, 2014; Gonzales et al. 2009; Jasti et al. 2007). Deg/ENaC channels are trimeric containing three similar subunits arranged to form a central conductive pore. Deg/ENaC subunits are intrinsic membrane proteins that span the membrane twice containing two transmembrane domains that separate a relatively large extracellular domain from short intracellular NH_4_- and COOH-termini. The large extracellular domain has complex secondary and tertiary structure, and goes through proteolytic posttranslational modification that is critical for proper channel function (Pacha et al. 1991; Hughey et al. 2004; Carattino et al. 2006; Bruns et al. 2007; Passero et al. 2012). The conductive pore of a Deg/ENaC channel is hour-glass in shape and formed by the symmetry-related second transmembrane (TM2) domains from each of the three component subunits. The selectivity filter of the Deg/ENaC pore lies near the intracellular mouth of the pore with the closing gate above this located at the bottom of the vestibule forming the extracellular mouth of the pore. Regulation of Deg/ENaC channel gating is complex and involves communication between extracellular and intracellular modulatory domains with the transmembrane domains that form the conductive pore.

The expression of ENaC in the apical membrane of principal cells is tightly regulated by cellular signaling. Aldosterone increases the residency time that ENaC spends within the apical membrane by inhibiting retrieval of the channel. Retrieval of ENaC from the apical membrane is promoted by ubiquitination of channel subunits by the ubiquitin ligase, Nedd4-2. Nedd4-2 through WW domains binds to PY motifs in the cytosolic COOH tails of channel subunits and ubiquitinates lysines in the cytosolic NH_2_-termini of these subunits. Aldosterone activates the kinase, Sgk1, which phosphorylates Nedd4-2 facilitating sequestration of this ubiquitin ligase by 14-3-3 proteins away from the channel (Snyder 2002; Bhalla et al. 2005). Thus, aldosterone increases ENaC activity through a disinhibition mechanism increasing the residency of the channel in the apical membrane.

The majority of disease-causing mutations in ENaC (*SCNN* genes) results in the abnormal expression of the channel within the apical plasma membrane of principal cells. They either result in the production of nonfunctional transcripts, misfolded subunits incompatible with functional oligomerization, or channel subunits lacking normal PY motifs. Recent findings have identified a few disease-causing mutations in ENaC, including the K106_S108delinsN and N530S mutations in *γ*ENaC and the S562P mutation in *α*ENaC, that may affect the biophysical and gating properties of the channel to influence activity (Strautnieks et al. 1996; Melander et al. 1998; Schaedel et al. 1999; Hiltunen et al. 2002; Riepe et al. 2009). This speculation arises primarily from the position of the affected residues within channel subunits and to a more limited extent from cursory functional analysis of channels harboring these mutations.

Identification of disease-causing mutations that affect the biophysical and gating properties of ENaC will be informative about the atomic movements and forces that control ENaC function as an ion channel. We hypothesized that disease-causing mutations in *γ*ENaC within the extracellular mouth of the pore and within finger domains recognized to influence gating; and within the selectivity filter domain of *α*ENaC would affect the biophysical and/or gating properties of the channel.

The current results demonstrate for the first time that the PHA1 and Liddle’s syndrome-causing mutations, K106_S108delinsN and N530S, respectively, in *γ*ENaC decrease and increase channel open probability. The PHA1 S562P mutation in *α*ENaC compromises channel function most likely by disturbing normal channel permeation and conduction by disordering the structure of the selectivity filter.

## Methods

### Reagents and cDNA

All reagents were from Sigma (St. Louis, MO) or Fisher (Pittsburg, PA) unless noted otherwise. The mammalian expression vectors encoding mouse *α*-, *β*-, and *γ*ENaC subunits have been described previously (Pochynyuk et al. 2007, 2009; Kucher et al. 2011). All mutagenesis was performed on the backbone of these plasmids by TOP Gene Technologies (Montreal, QC, Canada). Every plasmid encoding a mutant channel subunit was sequenced to insure proper incorporation of the expected mutation and to confirm sequence identity, orientation and reading-frame.

### Cell culture and transfection

Chinese Hamster Ovary (CHO) cells, from American Type Culture Collection, were used for heterologous expression of mENaC. CHO cells were cultured following the standard procedures: Cells were maintained at 37°C in 5% CO_2_ with medium containing DMEM + 10% FBS (Pochynyuk et al. 2007, 2009; Kucher et al. 2011). Recombinant mENaC was expressed in CHO cells via transfection with expression plasmids (0.3 *μ*g/subunit/9.6 cm^2^ plasmid cDNA) using the Polyfect reagent (Qiagen; Valencia, CA) following the manufacturer’s suggested protocol. Electrophysiological experiments were performed 48–72 h after transfection.

### Patch clamp electrophysiology

Whole-cell current recordings were made under voltage-clamp conditions as described previously (Pochynyuk et al. 2007, 2009; Kucher et al. 2011). Currents were filtered at 1 kHz and acquired at 2 kHz with an Axopatch 200B (Molecular Devices, Sunnyvale, CA) interfaced via a Digidata 1322A (Molecular Devices) to a PC running the pClamp 10.2 software suite (Molecular Devices). Cell capacitance, ∼9 pF for CHO cells, was routinely compensated. Pipette resistance was in the range 2–5 MΩ. Only recordings where access resistance and capacitance changed less than 10% over the course of the experiment were used. The extracellular bath solution was (in mmol/L) 150 NaCl, 1 CaCl_2_, 2 MgCl_2_, 10 HEPES (pH 7.4; ∼300 mOsm). For these studies, both asymmetrical and symmetrical intracellular pipette solutions containing (in mmol/L) 145 CsCl plus 5 NaCl, or 150 NaCl, respectively, and 2 MgCl_2_, 5 EGTA, 2 ATP, 0.1 GTP, and 10 HEPES (pH 7.4; ∼300 mOsm) were used. With asymmetrical solutions, currents were evoked with a 0.5 sec voltage ramp ranging from 60 to −100 mV from a holding potential of 40 mV. With symmetrical solutions, currents were evoked via a 20 mV voltage-step ranging from 100 to −100 mV from a holding potential of 0 mV.

Single-channel current recordings in outside-out patches were made under voltage-clamp conditions using an Axopatch 200B following standard procedures (Pochynyuk et al. 2007, 2009; Kucher et al. 2011). Currents were low-pass filtered at 100 Hz using an eight-pole Bessel filter (Warner Instr., Hamden, CT); digitized at 500 Hz and stored on a PC using the Digidata 1440A interface. Symmetrical extracellular bath and intracellular pipette solutions containing (in mmol/L) 150 LiCl, 1 CaCl_2_, 2 MgCl_2_, 10 HEPES (pH 7.4; ∼300 mOsm); and 150 LiCl, 2 MgCl_2_, 5 EGTA, 2 ATP, 0.1 GTP, and 10 mm HEPES (pH 7.4; ∼300 mOsm), respectively, were used. Currents were evoked via a 20 mV voltage-step ranging from 80 to −80 mV. Only patches containing five or fewer channels were used for analysis.

### Data analysis and presentation

Data are reported as mean ± SEM. Unpaired data were compared using a two-sided unpaired Student’s *t*-test or One-Way Analysis of Variance using the Student–Newman–Kuels post *hoc* test as appropriate. Proportions were compared using a *z*-test. The criterion for significance was *P* < 0.05. Macroscopic current–voltage (*I*–*V*) curves were normalized to instantaneous current at 100 mV hyperpolarized from the reversal potential. Single-channel current–voltage (*I*–*V*) relations were generated from unitary currents (*i*) defined by all-point amplitude histograms at different holding potentials from, at least, four independent experiments. Channel activity defined as *NP*_o_ was equal to *Σ*(*t*_1_ + 2*t*_2_ + …*it*_*i*_), where *t*_*i*_ is the fractional open time spent at each of the observed current levels. *P*_o_ was determined by dividing *NP*_o_ by the number of channels within a patch as established with all-point amplitude histograms. *P*_o_ was calculated over ≥30 sec for each data point. For presentation, some currents were subsequently software filtered at 40 Hz. For ease of presentation, all amino acids in figures and text are referenced to their position in the human ENaC subunits where *α*S562 in the human protein is homologous to S589 in the mouse protein; *γ*K106_S108N is identical in the human and mouse proteins; and *γ*N530 in the human protein is homologous to N536 in the mouse.

## Results

### PHA1 and Liddle’s Syndrome mutations cause differential changes in ENaC activity

**Figure 1:**
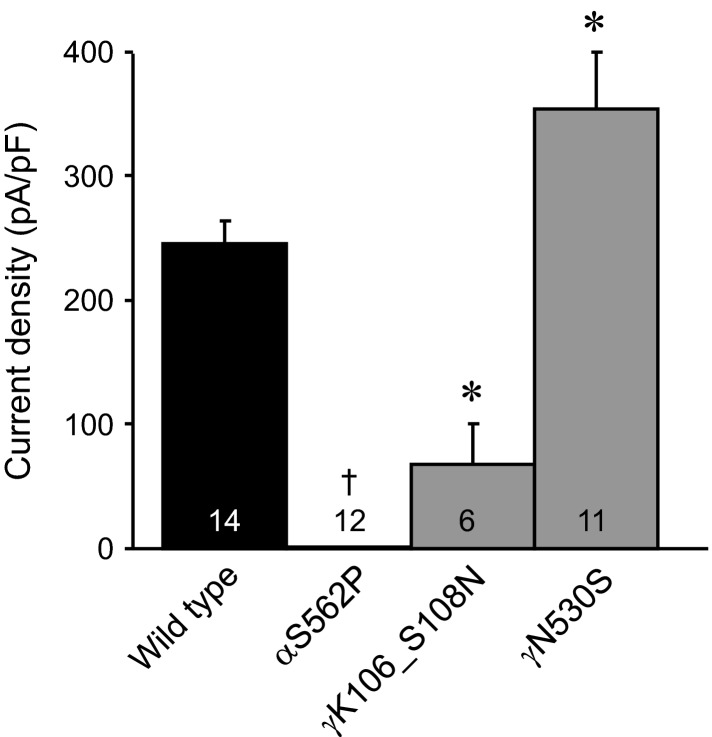
Disease-causing mutations affect ENaC activity. Summary graph of (10 μmol/L) amiloride-sensitive macroscopic Na^+^ current density at −80 mV for CHO cells in asymmetrical solutions expressing wild-type ENaC and ENaC harboring disease-causing mutations in either the *α* or *γ* subunit. The number of experiments for each group is indicated in each histogram. *Significant change versus wild-type ENaC. ^†^Incorporation of the S562P mutation into *α*ENaC resulted in a significant decrease in the proportion of cells that expressed ENaC currents.

Figure1 compares the steady-state current density at −80 mV of ENaC containing PHA1-causing mutations, including the S562P missense mutation in *α*ENaC and the K106_S108delinsN in *γ*ENaC; and the N530S missense mutation in *γ*ENaC associated with Liddle’s Syndrome with that of wild-type ENaC expressed in CHO cells. Introduction of K106_S108N into *γ*ENaC resulted in a significant decrease in macroscopic ENaC current density. In contrast, introduction of N530S into *γ*ENaC significantly increased macroscopic ENaC current density. Incorporation of the S562P mutation into *α*ENaC resulted in a significant decrease in the proportion of cells that expressed ENaC currents. No ENaC current was observed in any cell containing (wild-type *β* and *γ*ENaC plus) the S562P *α*ENaC mutant. These findings are consistent with previous studies showing that PHA1- and Liddle’s syndrome-causing mutations result in loss and gain of ENaC function, respectively (Snyder et al. 1995; Grunder et al. 1997; Hiltunen et al. 2002; Kucher et al. 2011).

### The PHA1-causing mutation, *γ*K106_S108N, decreases ENaC open probability and unitary conductance

**Figure 2:**
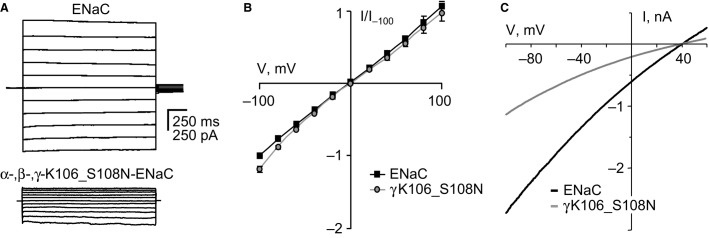
The PHA1 mutation, *γ*K106_S108N, decreases macroscopic ENaC current. (A) Representative families of macroscopic Na^+^ currents evoked by progressive voltage-steps (ranging from −100 to 100 mV in 20 mV increments from a holding potential of 0 mV) in voltage-clamped CHO cells in symmetrical solutions expressing wild-type (top) and mutant (bottom) ENaC. (B) Steady-state macroscopic current–voltage (*I*–*V*) relations for CHO cells expressing wild-type (black line) and mutant (gray line) ENaC. Current–voltage relations generated from experiments similar to that in (A) with currents normalized to instantaneous current at −100 mV. (C) Instantaneous macroscopic current–voltage (*I*–*V*) relations for voltage-clamped CHO cells expressing wild-type (black line) and mutant (gray line) ENaC in asymmetrical solutions. Currents evoked with a voltage ramp from 60 to −100 mV (from a holding potential of 40 mV) over a 0.5 sec trial period.

To reveal the mechanism that underlies loss and gain of function for these ENaC mutants, we used patch clamp electrophysiology to explore their biophysical and gating properties. Figure2A shows representative families of macroscopic Na^+^ currents in symmetrical NaCl solutions evoked by 20 mV voltage steps from 100 mV to −100 mV from a holding potential of 0 mV in cells expressing wild-type (top), and mutant (bottom) channels containing the K106_S108N substitution in *γ*ENaC. Mutant ENaC had significantly decreased macroscopic current. Figure2B shows corresponding (normalized) *I*–*V* relations for wild-type and mutant ENaC at steady state. As expected, wild-type ENaC had a linear *I*–*V* relation in symmetrical NaCl solutions. Similarly over the voltages probed, the *I*–*V* relation for ENaC containing the PHA1 mutation was linear in symmetrical solutions. In asymmetrical solutions, as shown in Figure2C, wild-type and mutant ENaC had reversal potentials (39 ± 0.5 and 38 ± 1.5 mV, respectively; *P* = 0.49) that were not different. Thus, analysis of macroscopic current failed to reveal the underlying cause for the decreased activity observed for this mutant ENaC.

**Figure 3:**
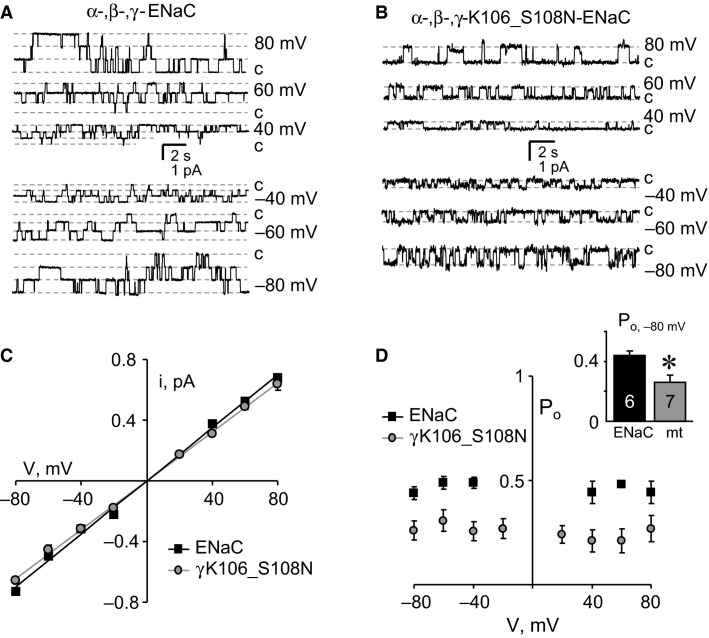
The PHA1 mutation, *γ*K106_S108N, decreases the open probability and unitary conductance of ENaC. Representative families of single-channel current traces from wild-type (A) and mutant (B) ENaC in an outside-out patch in symmetrical solutions stepped from 80 to −80 mV. Inward Na^+^ current is downwards with closed states noted with *c*. (C) Single-channel current–voltage (*I*–*V*) relations for wild-type (black squares) and mutant (gray circles) ENaC. Data are from experiments identical to that in (A & B); *n* ≥ 7 for each group. (D) Plot showing ENaC open probability (*P*_o_) as a function of voltage for wild-type (black squares) and mutant (gray circles) channels. Data are from experiments identical to that in 3A; *n* ≥ 6 for each group. The inset compares single-channel open probability at −80 mV for wild-type (black bar) and mutant (gray bar) ENaC. *Significant (*P* < 0.013) decrease versus wild-type.

We next studied wild-type ENaC, and ENaC containing the K106_S108N mutation at the single-channel level in excised, outside-out patches. Continuous current traces for typical wild-type and mutant ENaC at voltages ranging from 80 to −80 mV are shown in Figures3A and B, respectively. The *I*–*V* relations for mutant and wild-type ENaC shown in Figure3C, demonstrate that mutant channels had a slight but significant decrease in unitary conductance (*g *=* *8.00 ± 0.11 for mutant, and 8.73 ± 0.24 pS for wild-type; *P* = 0.03).

As documented in Figure3D, ENaC harboring the K106_S108N mutation also had a significantly decreased steady-state *P*_o_ at −80 mV compared to wild-type ENaC. This decrease in *P*_o_ was sustained across all voltages tested. These data are the first evidence that this PHA1-causing mutation decreases channel activity via changes in open probability and unitary conductance.

### The Liddle’s syndrome-causing mutation, *γ*N530S, increases ENaC open probability

**Figure 4:**
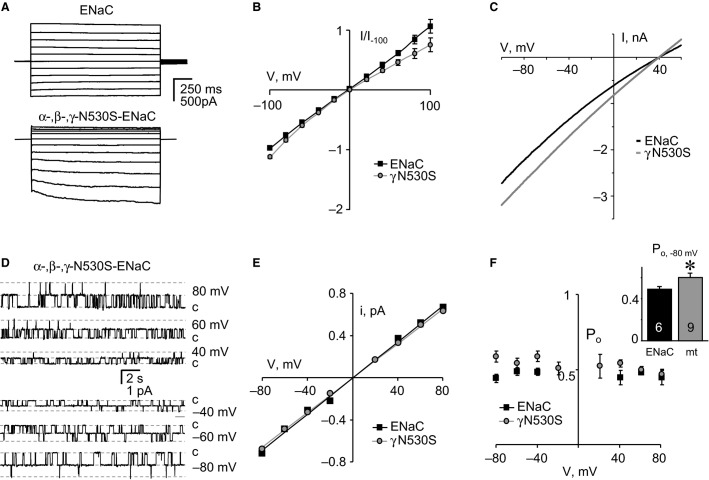
The Liddle’s Syndrome mutation, *γ*N530S, increases the open probability of ENaC. (A) Representative families of macroscopic Na^+^ currents evoked in voltage-clamped CHO cells expressing wild-type (top) and mutant (bottom) ENaC. All other experimental conditions are identical to Figure2A. (B) Steady-state macroscopic current–voltage (*I*–*V*) relations for CHO cells expressing wild-type (black line) and mutant (gray line) ENaC. Data are from experiments identical to that in (A). (C) Instantaneous macroscopic current–voltage (*I*–*V*) relations for voltage-clamped CHO cells expressing wild-type (black line) and mutant (gray line) ENaC in asymmetrical solutions. All other conditions are identical to Figure2C. (D) A representative family of single-channel current traces from mutant ENaC in an outside-out patch in symmetrical solutions stepped from 80 to −80 mV. Inward Na^+^ current is downwards with closed states noted with *c*. (E) Single-channel current–voltage (*I*–*V*) relations for wild-type (black squares) and mutant (gray circles) ENaC. Data are from experiments identical to that in (D); *n* ≥ 7 for each group. (F) Plot showing ENaC open probability (*P*_o_) as a function of voltage for wild-type (black squares) and mutant (gray circles) channels. Data are from experiments identical to that in (D); *n* ≥ 6 for each group. The inset compares single-channel open probability at −80 mV for wild-type (black bar) and mutant (gray bar) ENaC. *significant (*P* < 0.01) increase versus wild-type.

Figure4A shows representative families of macroscopic Na^+^ currents in symmetrical NaCl solutions for wild-type (top) and mutant (bottom) channels containing the N530S substitution in *γ*ENaC. Mutant ENaC had significantly increased macroscopic currents at the hyperpolarizing potentials. Figure4B shows corresponding (normalized) *I*–*V* relations for wild-type and mutant ENaC at steady state. Both wild-type and mutant ENaC had linear *I*–*V* relations in symmetrical NaCl solutions. In asymmetrical solutions, as shown in Figure4C, wild-type and mutant ENaC had reversal potentials (39 ± 0.5 and 42 ± 1.5 mV, respectively; *P* = 0.13) that were not different. Single-channel current traces for mutant ENaC are shown in Figure4D. As demonstrated by the resulting *I*–*V* relations shown in Figure4E, the unitary conductance of this mutant (*g *=* *8.30 ± 0.10 pS; *P* = 0.15) was not different from that of wild-type ENaC. As documented in Figure4F, *P*_o_ was significantly greater at hyperpolarizing potentials in ENaC harboring the *γ*N530S mutation compared to the channel containing all wild-type subunits. The potentials at which *P*_o_ was greater in the mutant channel are close to those which the channel would face across the apical membrane in native polarized distal nephron principal cells. These data are the first direct evidence that this Liddle’s syndrome-causing mutation increases channel activity by increasing open probability.

## Discussion

The current studies demonstrate that two disease-causing mutations in *γ*ENaC, K106_S108delinsN, PHA1, and N530S, Liddle’s syndrome, decrease and increase channel *P*_o_, respectively. The prior mutation also decreases unitary channel conductance. Moreover, PHA1 mutations affecting S562 in *α*ENaC likely decrease channel activity by influencing the biophysical properties of the channel. In this latter case, mutation of S562 likely results in a nonfunctional channel with a disorganized conduction pathway.

**Figure 5:**
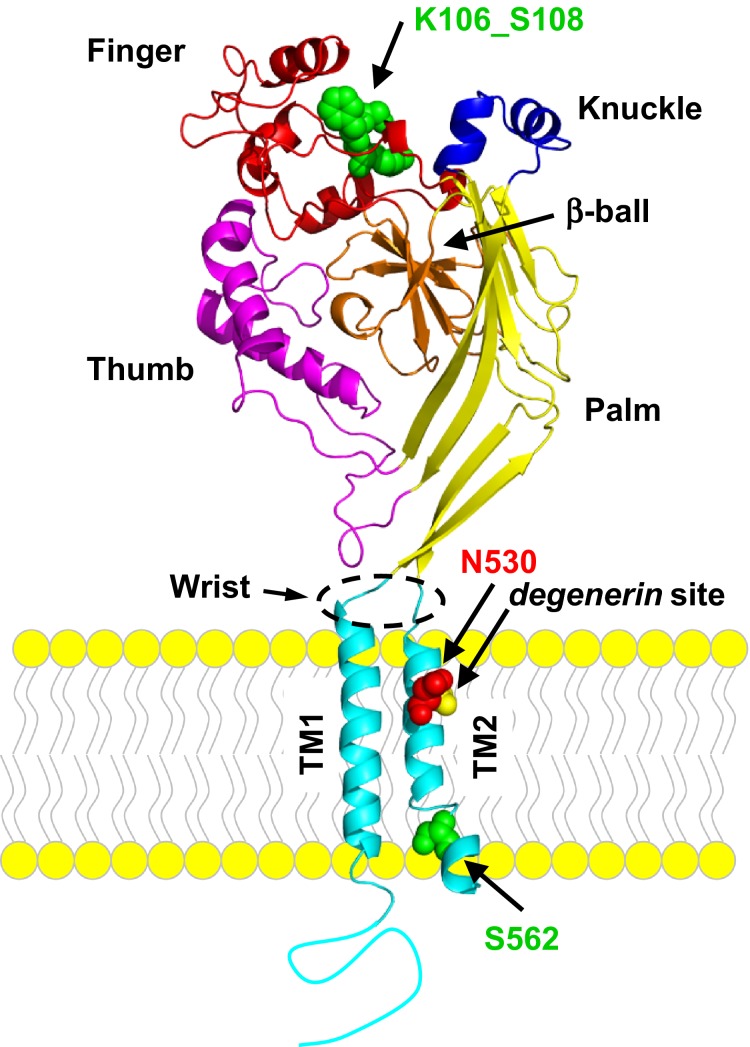
Secondary and tertiary structure of an archetype Deg/ENaC subunit. This model of an ENaC subunit shows the position of the residues investigated in this study. This model is based upon the cASIC1 structure as published by the Gouaux laboratory (Jasti et al. 2007; Gonzales et al. 2009; Baconguis et al. 2013). In this model, major tertiary structures, including the transmembrane domains (TM1 & TM2; light blue), the wrist linker, and palm (yellow), thumb (pink), finger (red), knuckle (dark blue), and *β*-ball (orange) domains are indicated. The residues mutated in these studies are noted and shown with space-filling with gain-of-function mutations red and loss-of-function green. The *degenerin* position is also shown space-filled (yellow).

The TM2s of the three symmetry-related subunits that comprise heterotrimeric ENaC, *α*, *β,* and *γ*, form the conduction pore of the channel through the plasma membrane (Sheng et al. 2001a,b; Takeda et al. 2007). As shown in the model presented in Figure5 of an archetype ENaC subunit, which is based upon the chicken ASIC1 crystal structure as solved by the Gouaux laboratory (Jasti et al. 2007; Gonzales et al. 2009; Baconguis and Gouaux 2012; Baconguis et al. 2013, 2014), N530 is located in TM2 of the *γ*ENaC subunit near the extracellular mouth of the pore. Most Deg/ENaC proteins, excluding all ENaC subunits, have a conserved aspartic acid at the homologous position (D433 in cASIC1). Every ENaC subunit has an asparagine at this site. Similar to ASIC, ENaC is thought to be in a nonconducting closed state when there is constriction of the pore at the channel gate with relaxation of this constriction during channel opening allowing permeation and conduction (Gonzales et al. 2009; Baconguis and Gouaux 2012; Baconguis et al. 2013, 2014). As revealed in the crystal structure, the gate of cASIC1 is at the bottom of the extracellular vestibule of the pore which is defined by the aspartic acids in positions homologous to N530 (Jasti et al. 2007; Gonzales et al. 2009; Baconguis and Gouaux 2012; Baconguis et al. 2013, 2014). Functional studies demonstrate that D433 (homologous position as N530) along with G432 form the pore’s closing gate in ASIC1 (Li et al. 2011). Because the crystal structure of heterotrimeric ENaC has not been resolved yet, the steric and mechanistic importance of having an asparagine rather than an aspartic acid at the position homologous to 530 is not fully clear but may contribute to the observation that dissimilar to ASIC, ENaC gates constitutively and does not desensitize possibly due to some destabilization of the closing gate.

Deg/ENaC proteins were first identified and named from work performed in the *C. elegans* model system. In *C. elegans*, expression of *me*c and *deg* genes in touch receptors are required for mechanosensation (Chalfie and Wolinsky 1990; Driscoll and Chalfie 1991; Hong and Driscoll 1994). Some *mec* and *de*g genes, including *mec-4* and *deg-1*, are similar in sequence, and code for what are now understood to be Deg/ENaC channel subunits. Mutation at the *degenerin* site (A713, referred to as A442 in the original publications) in *mec-4* causes degeneration of touch receptors due to pathological cell swelling resulting from constitutive activation of a cation selective influx pathway (Driscoll and Chalfie 1991; Hong and Driscoll 1994). *Degenerin* mutations as originally identified in *mec-4* are thought to prevent the channel from closing due to steric hinderance. Mutations at this site are dominant consistent with them resulting in a gain-of-function of the channel. The *degenerin* site is one position upstream from N530 in *γ*ENaC and at the position homologous to G432 in ASIC1. As discussed above, residues at both of these positions contribute to the pore’s closing gate. This agrees with a mechanism whereby *degenerin* mutations increase *P*_o_ by hindering closing of the channel through destabilization of the closing gate. It is reasonable to suggest that the Liddle’s mutation, N530S, studied here results in a similar manifestation where channel *P*_o_ increases as a result of disruption of channel closure due to destabilization of the closing gate. Our findings that *P*_o_ is increased in the N530S mutation are consistent with such a mechanism causing inappropriate ENaC activity resulting in the Liddle’s phenotype associated with this mutation in humans (Melander et al. 1998; Hiltunen et al. 2002).

Functional and structural studies agree that the gate and selectivity filter of ENaC are physically separated from each other (Li et al. 2011; Baconguis et al. 2014). Compelling evidence is consistent with S562 being within the selectivity filter of ENaC (Sheng et al. 2001a,b; Takeda et al. 2007). Homologous series (S445 in cASIC1) at this position are highly conserved across all Deg/ENaC subunits and are recognized to contribute critical structure to the selectivity filter of channels formed by these proteins. Substitution of this serine causes a fatal structural disruption of the pore producing a channel no longer able to conduct. Moreover, substitution of this serine in *mec-4* that also harbors the *degenerin* mutation blocks neurodegeneration of touch receptors in *C. elegans* (Hong and Driscoll 1994). The current findings are consistent with channels containing the PHA1 mutation S562P in the *α*ENaC subunit being unable to conduct. We propose that the S562L mutation of *α*ENaC, which also causes PHA1 (Schaedel et al. 1999), results in a similar nonfunctional channel unable to conduct due to a disrupted permeation pathway. The crystal structure of cASIC1 shows that having a serine at the position homologous to S562 is required for proper trigonal antiprism steric coordination of the permeant ion within the pore (Jasti et al. 2007; Gonzales et al. 2009; Baconguis and Gouaux 2012; Baconguis et al. 2014). Based upon this, we speculate that substitution of this critical serine with any larger, and possibly smaller residue, fatally disrupts the rigid steric requirements for permeation leading to a nonfunctional conduction pathway. With this in mind, we speculate further that substitution of S562 in *α*ENaC and its homologous residues in other channel subunits with any amino acid will always result in a nonfunctional channel manifesting in disease.

The molecular mechanism whereby the K106_S108delinsN mutation leads to decreases in channel activity is more difficult to conceive. As shown here, this mutation both decreases *P*_o_ and unitary channel conductance. Thus, it must through some means affect both gating as well as impact how quickly Na^+^ transits through the pore. The simplest way to rectify both observations is to suggest that by destabilizing normal intra- and/or inter-subunit interactions, this mutation ultimately disrupts the outer vestibule of the pore to include the region around the closing gate (near N530 in *γ*ENaC). Such disruption then would have to stabilize the closing gate to exert the opposite action on *P*_o_ as the N530S mutation.

The residues forming the extracellular vestibule of the pore create a reservoir that has a large negative electrostatic potential (Gonzales et al. 2009; Baconguis and Gouaux 2012; Baconguis et al. 2013, 2014). This negatively charged reservoir attracts cations, to include Na^+^, and consequently, has been argued to make a substantial contribution to the conductance of Deg/ENaC channels by concentrating permeant ions near the mouth of the permeation pathway. Any disruption within channel subunits that destabilizes this reservoir or influences its electrostatic potential could conceivably affect conductance. The confluence of this reservoir with the channel gate creates the ideal spatial arrangement whereby a destabilization of structure at a single site could simultaneously affect conductance and *P*_o_.

A slight variation on this possible mechanism could be that this mutation by ultimately influencing structure near the channel gate introduces a novel energy barrier to permeation or increases the magnitude of an existing energy barrier rather than affecting the cation reservoir. The region near the closing gate of the channel is narrow. Any mutation that ultimately influences this region of the channel to further narrow the pore could potentially increase the energy required to permeate through the pore and thus, slow transit and decreases conductance. Importantly, all other rationalizations of potential mechanism that do not include ultimate action at the bottom of the extracellular vestibule of the pore near the closing gate would entail the K106_S108delinsN mutation exerting two independent actions through at least two distinct effects on structure, one that influences gating and another conductance.

As shown in Figure5, K106_S108 residues are located between the *β*2 beta sheet and *α*1 alpha helix common to all Deg/ENaC subunits. The *β*2 beta sheet contributes to the central *β*-ball that is found between the palm and thumb structures of Deg/ENaC subunits (Jasti et al. 2007). The *α*1 alpha helix defines the beginning of the finger domain of Deg/ENaC subunits. The greatest sequence variation among Deg/ENaC subunits is found in the finger regions of these proteins (Stockand et al. 2008). Thus, this is the area that provides the greatest distinction to each channel subunit. It is recognized that the juxtaposition of the thumb, finger, palm and *β*-ball domains of channel subunits influences gating (Jasti et al. 2007; Gonzales et al. 2009; Baconguis and Gouaux 2012; Baconguis et al. 2013, 2014). This is so because the thumb and palm domains are coupled to the wrist as it is hinged to the pore at the confluence of the cation attracting reservoir and channel closing gate. It is reasonable to suggest that the K106_S108delinsN mutation disrupts important structure in the finger region of this subunit affecting its juxtapositioning on thumb domains, which would impact the wrist region of the channel and consequently the gate and cation reservoir to influence both *P*_o_ and conductance.

Not necessarily distinct from this possible mechanism, the position of the K106_S108delinsN mutation in the finger domain likely would also influence the impact of posttranslational proteolytic cleavage of this subunit. Furin, prostasin, plasmin, and various elastases can cleave ENaC subunits (Pacha et al. 1991; Hughey et al. 2004; Carattino et al. 2006; Bruns et al. 2007; Passero et al. 2012). These enzymes cleave the *γ*ENaC subunit in the finger domain a little downstream of the K106_S108 residues. Cleavage influences gating possibly by releasing or relaxing an inhibitory domain within the finger region (Carattino et al. 2008; Passero et al. 2010, 2012), which is ultimately translated to the channel gate via intra- and inter-subunit interactions of the finger domain with thumb domains (Maarouf et al. 2009; Shi et al. 2011; Baconguis and Gouaux 2012; Baconguis et al. 2013, 2014). A mutation, such as the K106_S108delinsN, which would affect the position of these cleavage sites and thus, how the cleaved finger domain of *γ*ENaC interacts with thumb domains would be expected to impact *P*_o_. As demonstrated here, it does. Importantly, such a mechanism would retain changes in structure ultimately at the region of the channel near the confluence of the gate and extracellular vestibule of the pore. Thus, we propose that effects of the K106_S108delinsN mutation on the finger region likely involving influences on channel cleavage as translated through the thumb and wrist domains ultimately influence gating and conductance by affecting the area of the pore around the closing gate.

## Conflict of Interest

None declared.
